# Elastographic Changes in Cervical Muscle Following Combined Radial Extracorporeal Shockwave Therapy and Orthopedic Manual Therapy: A Randomized Controlled Trial

**DOI:** 10.3390/jcm15124767

**Published:** 2026-06-19

**Authors:** Carlos López-Moreno, Javier Conde-Pipó, Antonio Martínez-Amat, Alexander Achalandabaso-Ochoa

**Affiliations:** 1 Department of Health Sciences, San Juan de Dios School of Nursing and Physical Therapy, Comillas Pontifical University, 28350 Madrid, Spain; clopez@comillas.edu; 2 San Juan de Dios Foundation, 28016 Madrid, Spain; 3 Department of Health Sciences, Faculty of Health Sciences, University of Jaén, 23071 Jaén, Spain; amamat@ujaen.es (A.M.-A.); aaochoa@ujaen.es (A.A.-O.); 4 Health Science and Nutrition Research (HSNR, CTS-1118), Department of Nutrition and Food Science, School of Pharmacy, University of Granada, 18071 Granada, Spain

**Keywords:** extracorporeal shockwave therapy, shear wave elastography, manual therapy, randomized controlled trial, neck pain

## Abstract

**Background:** Non-specific neck pain is associated with altered muscle mechanical properties, including increased stiffness. Radial extracorporeal shockwave therapy (rESWT) and orthopedic manual therapy (OMT) are commonly used interventions, although their combined effects on cervical muscle stiffness remain unclear. This study aimed to evaluate the short-term and within-session effects of adding rESWT to OMT on cervical muscle stiffness measured by means of shear wave elastography (SWE) in individuals with non-specific neck pain. **Methods:** A randomized controlled trial was conducted including 24 participants (mean age 34.36 years) allocated to an intervention group (IG, *n* = 12) or a control group (CG, *n* = 12). The IG received a combined protocol of rESWT (1500 impulses per point at 10 Hz, 2–4 bar) and OMT based on the Maitland concept, while the CG received OMT alone. Primary outcomes included cervical muscle stiffness assessed via SWE expressed in meters per second (m/s) and kilopascals (kPa). Secondary outcomes were pain intensity (VAS), pressure pain threshold (PPT), cervical range of motion (ROM), and shoulder elevation strength (SES). Treatment effects were estimated using ANCOVA adjusted for baseline values. **Results:** The combined intervention was associated with greater reductions in cervical muscle stiffness compared with the control group, with significant decreases in SWE values (m/s: β = −1.27, *p* < 0.001; kPa: β = −27.97, *p* < 0.001). Pain intensity was also reduced (β = −2.12, *p* = 0.012), while PPT increased (β = 18.84, *p* = 0.024). Improvements were observed in cervical extension ROM (β = 10.30, *p* = 0.014) and right SES (β = 3.85, *p* = 0.044). No significant differences were found for other ROM variables or left SES. **Conclusions:** The addition of rESWT to OMT was associated with greater short-term improvements in cervical muscle stiffness, pain intensity, and mechanical sensitivity compared with OMT alone in individuals with non-specific neck pain. However, these findings should be interpreted with caution due to the study limitations.

## 1. Introduction

Non-specific neck pain is one of the most prevalent musculoskeletal disorders worldwide and represents a major cause of reduced quality of life and disability [1]. Its multifactorial etiology, involving mechanical, neuromuscular, and psychosocial factors, contributes both to the development and persistence of symptoms [2,3]. Among the proposed pathophysiological mechanisms, altered cervical motor control and increased muscle tissue stiffness have been identified as relevant factors associated with pain and restricted cervical spine movement [4,5].

Recent advances in ultrasound imaging have enabled the use of shear wave elastography (SWE), a technique that provides quantitative assessment of tissue stiffness expressed in meters per second (m/s) or kilopascals (kPa) [6]. Increased stiffness of the cervical musculature has been associated with higher pain intensity, impaired neuromuscular control, and reduced functional performance in individuals with neck pain [4,7]. However, evidence on the effects of conservative therapeutic interventions on these mechanical properties remains limited.

Orthopedic manual therapy (OMT) is widely used in the management of neck pain and has demonstrated beneficial effects on pain and function through mechanical and neurophysiological mechanisms [8]. Radial extracorporeal shockwave therapy (rESWT) has also gained interest as a non-invasive intervention capable of modulating pain, enhancing tissue perfusion, and reducing myofascial stiffness [9,10]. Experimental and clinical studies suggest that rESWT may promote angiogenesis, improve local metabolic conditions, and modulate nociceptive mediators such as substance P and calcitonin gene-related peptide (CGRP), contributing to analgesic effects and functional recovery [10,11,12,13]. Although both interventions have shown promising results independently, the potential synergistic effects of combining rESWT with OMT on cervical muscle mechanical properties remain poorly investigated. Most studies have primarily focused on subjective outcomes such as pain or disability, while objective biomechanical changes in muscle tissue have received less attention.

Therefore, the aim of this study was to evaluate the effects of a four-week combined intervention of rESWT and OMT on cervical muscle stiffness measured by means of shear wave elastography in individuals with non-specific neck pain. Secondary aims included changes in pain intensity, pressure pain threshold, cervical range of motion, and shoulder elevation strength, as well as the persistence of treatment effects one month after the intervention.

## 2. Materials and Methods

### 2.1. Study Design and Ethics

This study was a prospective, controlled, randomized clinical trial designed to evaluate the short-term and within-session effects of adding rESWT to OMT in individuals with non-specific neck pain. Participants were randomly allocated to either a control group (CG), who received OMT alone, or an intervention group (IG), who received OMT combined with rESWT. Randomization was performed using a block randomization procedure, consisting of blocks of two participants repeated until the target sample size was reached. Allocation concealment was ensured through the use of sealed opaque envelopes, which were available to the investigator responsible for participant assignment. Due to the nature of the interventions, blinding of participants and therapists was not feasible. Outcome assessments were conducted by the same examiner, who was not blinded to group allocation.

The intervention was carried out over a four-week treatment period, consisting of one session per week. Repeated measurements were obtained before and after each treatment session to assess within-session changes. Additional assessments were performed at baseline, immediately after the intervention period, and at a one-month follow-up to evaluate the persistence of treatment effects.

All study protocols and procedures were conducted in accordance with the Declaration of Helsinki and approved by the Ethics Committee of the Hospital Clínico San Carlos (protocol code 20/766-EC_X, 04/01/2021). This randomized clinical trial was prospectively registered at ClinicalTrials.gov (Identifier: NCT04758065). The participants were fully informed about the study’s objectives and methods, and each provided written informed consent before participation.

### 2.2. Participants

A total of 24 participants were included in the study and randomly allocated to either the control group (*n* = 12; 3 women [25%]; age 32.43 ± 15.59 years; Body mass indes (BMI) 24.93 ± 4.07 kg/m^2^; 41.67% active) or the intervention group (*n* = 12; 9 women [75%]; age 36.29 ± 10.98 years; BMI 21.11 ± 2.54 kg/m^2^; 50% active). Participants were recruited through consecutive sampling from individuals presenting with cervical pain at collaborating clinical centers. Although this recruitment strategy reflects routine clinical practice, it may introduce a potential risk of selection bias.

Eligible participants were adults aged between 18 and 59 years with a clinical diagnosis of non-specific neck pain. Consistent with current clinical practice guidelines and previous studies, non-specific neck pain was defined as cervical pain for which no specific structural or pathological cause could be identified through clinical examination and medical assessment [14,15,16]. The diagnosis was established through clinical history and physical examination and required the absence of signs or symptoms indicative of specific cervical disorders, including radiculopathy, neurological disease, inflammatory conditions, vertebral fractures, previous cervical trauma, congenital spinal abnormalities, infection, or neoplastic disease. Participants were excluded if they had a history of spinal surgery, neurological disorders, fibromyalgia, or were receiving concurrent physical therapy for the same condition. Information regarding medication use (e.g., analgesics, muscle relaxants, or antidepressants) was not systematically recorded or controlled during the study period, which may have influenced the observed outcomes.

### 2.3. Intervention Protocol

Treatment sessions were conducted weekly during the four-week intervention period. Both groups received a standardized 15-min session of OMT consisting of central postero-anterior mobilizations of the cervico-thoracic spine according to the Maitland concept, applied to painful or hypomobile segments [17]. The IG additionally received rESWT applied to painful points identified in the cervical musculature. Radial shockwave therapy was delivered using a clinical device designed for musculoskeletal applications. The treatment was applied directly over the identified trigger points using a protocol consisting of 1500 impulses per point, delivered in three cycles of 500 impulses, with a frequency of 10 Hz. The applied pressure ranged between 2 and 4 bar, corresponding to an estimated energy flux density of approximately 0.10–0.20 mJ/mm^2^, according to manufacturer specifications. The treatment parameters were selected according to previously published ESWT protocols used in musculoskeletal disorders and myofascial pain conditions [18,19,20].

Treatment intensity was individually adjusted to remain just below each participant’s pain threshold, ensuring tolerability while maintaining a clinically relevant stimulus [21,22]. The treatment schedule was selected based on previously described protocols in the literature. Current evidence suggests that rESWT should be applied at weekly intervals using the highest energy flux density tolerated by the patient to optimize therapeutic effects [20].

### 2.4. Outcome Measures

Several clinical and biomechanical variables were assessed to evaluate the effects of the intervention. All measurements were performed by the same trained examiner to minimize inter-observer variability.

- Cervical muscle stiffness was assessed using shear wave elastography (SWE). Participants were positioned in the prone position, and the ultrasound probe was placed over the previously identified pressure-sensitive point, perpendicular to the skin surface and aligned with the muscle fibers. The same previously identified pressure-sensitive point was assessed throughout all measurement sessions to ensure consistency between evaluations. Three consecutive measurements were obtained, and stiffness values were recorded in meters per second (m/s) and kilopascals (kPa) within a 1-cm diameter region of interest positioned at the center of the elastography window. The mean of the three measurements was used for statistical analysis [23].
- Pain intensity was assessed using the visual analogue scale (VAS), a widely used instrument consisting of a 10-cm horizontal line ranging from 0 (no pain) to 10 (worst imaginable pain) [24].
- Pressure-sensitive points were first identified through a scanning procedure using a radial pressure wave device (Masterpuls MP100 Ultra, Storz Medical AG, Tägerwilen, Switzerland) with a D20-S applicator (2–4 bar; 15 Hz). Participants indicated the locations where pressure elicited pain, and these points were marked on the skin to ensure consistent assessment before and after the intervention. The pressure pain threshold (PPT) was subsequently measured using a digital pressure algometer with the participant in the prone position. Three consecutive measurements were obtained with a 30-s interval, and the mean of the three trials was used for statistical analysis [25].
- Active cervical range of motion (ROM) was assessed using the EBI 5.0 motion analysis device (Retiatech S.L., Madrid, Spain). Participants were seated with their arms relaxed alongside the body. Two markers were placed on the skin: one at the occipital region and another between the second and third thoracic vertebrae. Participants performed active flexion, extension, lateral inclinations, and rotations of the cervical spine. Each movement was performed three times, and the mean value of the three repetitions was used for analysis [26].
- Shoulder elevation strength (SES) was assessed using a linear encoder system (T-Force Dynamic, Ergotech Consulting S.L., Mucrica, Spain). Participants were seated with the evaluated arm alongside the body, elbow extended, and the shoulder and forearm in a neutral position. The encoder was positioned vertically below the hand, and the participant performed a maximal shoulder elevation movement upon the evaluator’s signal. The test was repeated three times with a 10-s rest interval, and the mean value of the three repetitions was used for analysis [27].
- Physical activity level was assessed using the Spanish version of the Rapid Assessment of Physical Activity questionnaire (RAPA), a validated and user-friendly screening tool originally developed for adults. The instrument consists of seven items with yes/no response options, allowing rapid classification of participants according to their habitual physical activity level [28].

### 2.5. Data Collection Procedure

For each treatment session, outcome variables were recorded immediately before and after the intervention, allowing the calculation of within-session changes for each week of treatment. These acute responses were evaluated across the four treatment sessions. In addition, baseline measurements were compared with post-treatment and one-month follow-up values to assess the overall effectiveness of the intervention and the persistence of treatment effects over time.

### 2.6. Statistical Analysis

Descriptive statistics were calculated for all variables and expressed as mean and standard deviation (SD) for continuous variables and frequencies and percentages for categorical variables. Between-group differences in within-session changes across treatment weeks were assessed using independent-samples statistical tests. Changes from baseline to post-treatment and from post-treatment to follow-up were also compared between groups. Given the observed baseline differences between groups, adjusted treatment effects were examined using analysis of covariance (ANCOVA), controlling for baseline values. Longitudinal changes across the intervention period were assessed using linear mixed models to evaluate group × time interactions. Effect sizes were calculated using Hedges’ g to estimate the magnitude of between-group differences. Associations between cervical muscle stiffness, pain intensity, and pressure pain threshold were explored using Spearman correlation coefficients (ρ). As a sensitivity analysis, *p*-values from the ANCOVA models were additionally adjusted for multiple comparisons using the Benjamini–Hochberg false discovery rate procedure. Statistical significance was set at *p* < 0.05. All analyses were conducted using R version 4.0.2 (R Foundation for Statistical Computing, Vienna, Austria). Data visualization was performed using the ggplot2 package (version 3.4.2).

## 3. Results

**Figure 1:**
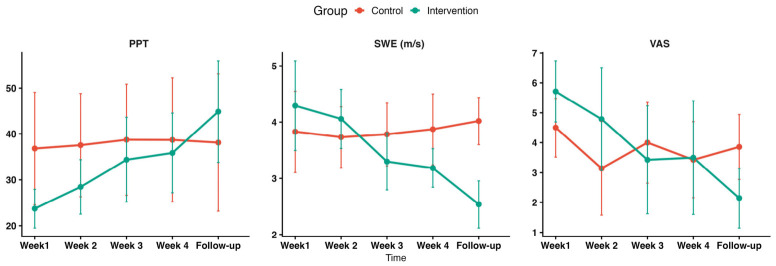
Longitudinal changes in primary outcomes.

Table 1 shows the immediate within-session changes across the four treatment weeks. The IG exhibited greater acute improvements than the CG, particularly in cervical muscle stiffness and pain-related outcomes. Reductions in SWE and increases in PPT were consistently greater in the IG across weeks. Pain intensity (VAS) decreased more in the IG during the first two treatment weeks, whereas no significant between-group differences were observed thereafter. Changes in cervical ROM and SES were generally small and not statistically different between groups, except for right lateral flexion after week 4 (*p* = 0.011). These within-session changes were consistent with the longitudinal trends observed across the intervention period, as illustrated in Figure 1.

Changes from baseline to post-treatment are presented in Table 2, including both absolute and relative changes for each group. Overall, the IG group demonstrated larger relative improvements than the CG, particularly in cervical muscle stiffness and pain intensity. SWE decreased by 37.5% (m/s) and 60.7% (kPa) in the IG compared with minimal changes in controls (*p* ≤ 0.007). Pain intensity (VAS) decreased by 67.1% in the IG versus 35.0% in the CG (*p* = 0.009). Cervical extension ROM increased by 21.4% in the IG (*p* = 0.025), whereas other outcomes did not show significant between-group differences.

**Figure 2:**
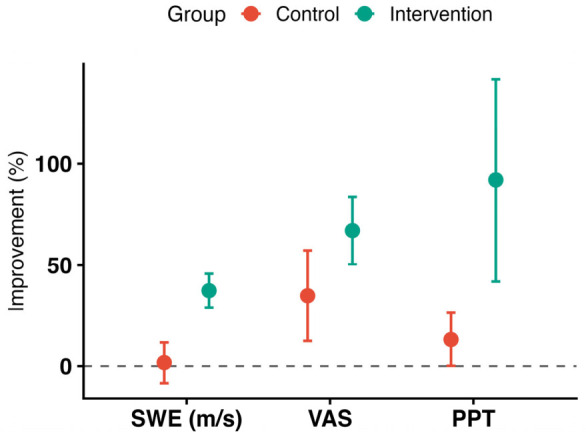
Change from baseline in primary outcomes.

The relative improvements in the main outcomes are illustrated in Figure 2, which summarizes the percentage change from baseline to post-treatment for cervical muscle stiffness, pain intensity, and pressure pain threshold. The IG showed larger improvements across all three outcomes compared with the CG.

Persistence of treatment effects from post-treatment to the 1-month follow-up is shown in Table 3. Overall, changes were small and not statistically different between groups for most outcomes. Cervical muscle stiffness and pain intensity remained relatively stable in the IG, whereas the CG showed slight increases in SWE and VAS values during follow-up. Although none of the between-group comparisons reached statistical significance, several outcomes showed moderate-to-large effect sizes, suggesting a tendency toward better maintenance of treatment effects in the intervention group.

**Figure 3:**
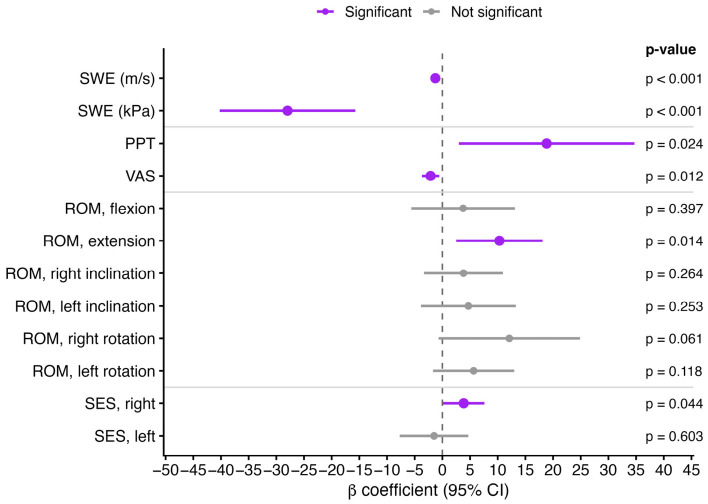
Adjusted treatment effects estimated using ANCOVA for clinical, biomechanical, and elastography outcomes, expressed as β coefficients with 95% confidence intervals. Significant effects are based on Benjamini–Hochberg false discovery rate-adjusted *p*-values.

The adjusted treatment effects estimated via ANCOVA are presented in Table 4 and illustrated in Figure 3. Between-group comparisons were adjusted for baseline values, and *p*-values were corrected for multiple comparisons using the Benjamini–Hochberg false discovery rate procedure (*p*FDR). Significant between-group differences were observed for cervical muscle stiffness, pain intensity, and pressure pain threshold. The intervention group showed lower SWE values expressed in m/s and kPa (both *p*FDR < 0.001), lower VAS scores (*p*FDR = 0.024), and higher PPT values (*p*FDR = 0.024) compared with the control group. No significant between-group differences were observed for cervical range of motion or shoulder elevation strength after *p*FDR correction.

Linear mixed models confirmed a different longitudinal evolution between groups for cervical muscle stiffness and pain-related outcomes, consistent with the trends shown in Figure 1. Significant group × time interactions were observed for SWE (m/s) (F = 12.051, *p* < 0.001), SWE (kPa) (F = 10.651, *p* < 0.001), PPT (F = 7.209, *p* < 0.001), and VAS (F = 4.017, *p* = 0.007). Significant interactions were also found for ROM extension (*p* = 0.001) and ROM right rotation (*p* = 0.004).

Spearman correlation analyses were performed to explore the relationships between cervical muscle stiffness, pain intensity, and pressure pain threshold within each group. In the intervention group, a moderate positive correlation was observed between SWE (m/s) and VAS (ρ = 0.551, *p* = 0.002), indicating that higher cervical muscle stiffness was associated with greater pain intensity. No significant correlations were found between SWE and PPT or between VAS and PPT in this group. In the control group, SWE (m/s) showed a moderate negative correlation with PPT (ρ = −0.404, *p* = 0.033), suggesting that greater muscle stiffness was associated with lower pressure pain thresholds. No other significant associations were observed.

## 4. Discussion

The present randomized clinical trial investigated whether adding rESWT to OMT could improve cervical muscle stiffness and clinical outcomes in individuals with non-specific neck pain. The main finding of this study was that the combined intervention was associated with greater reductions in cervical muscle stiffness measured via shear wave elastography (SWE), together with improvements in pain intensity and pressure pain threshold compared with OMT alone. These findings suggest that rESWT may enhance the therapeutic effects of manual therapy when incorporated into multimodal conservative management strategies for cervical pain. This interpretation is supported by recent evidence indicating that rESWT has been identified as a potentially beneficial physical modality in neck pain rehabilitation, ranking among the most effective interventions in a network meta-analysis including 34 randomized trials and more than 2000 patients [29].

A relevant finding of the present study is the objective reduction in cervical muscle stiffness. Increased stiffness has been associated with persistent pain, altered motor control, and impaired mechanical behavior of cervical musculature [30]. In this context, SWE provides clinically meaningful information beyond subjective pain scales by allowing direct quantification of tissue mechanical properties. Previous elastography studies have shown that myofascial trigger points exhibit higher stiffness than surrounding muscle tissue [6,31], supporting the relevance of stiffness assessment in cervical myofascial pain.

The present findings are consistent with previous research evaluating the effects of shockwave therapy on myofascial tissues. Reductions in tissue stiffness measured via elastography have been reported following focused rESWT in patients with upper trapezius myofascial pain syndrome, together with improvements in pain and disability [31]. Similar decreases in muscle stiffness have also been observed after radial shockwave therapy in patients with treatment-resistant trigger points [23]. Comparable changes in muscle mechanical behavior have been reported in other musculoskeletal conditions treated with rESWT, suggesting that shockwave therapy may induce measurable modifications in muscle tone and viscoelastic properties [32]. In line with this interpretation, the positive association observed in our sample between muscle stiffness and pain intensity supports the hypothesis that alterations in muscle mechanical properties may contribute to symptom severity rather than representing a simple epiphenomenon.

The clinical improvements observed in pain intensity and pressure pain threshold are also in line with previous randomized studies of rESWT in cervical and shoulder girdle myofascial pain. Repeated rESWT sessions have been shown to significantly reduce pain intensity and increase pressure pain threshold in patients with upper trapezius myofascial pain, whereas control interventions produced minimal changes [33]. Likewise, comparative trials have reported improvements in pain, disability, and quality-of-life outcomes after repeated treatment sessions, suggesting a cumulative therapeutic effect [32].

Evidence from comparative trials and meta-analyses provides additional context for these findings. Systematic reviews have consistently shown that rESWT significantly improves pain intensity and pressure pain threshold in myofascial pain syndrome, although pooled effects on disability remain less consistent [33,34]. Head-to-head comparisons with other commonly used interventions indicate that rESWT produces clinical improvements comparable to treatments such as manual therapy, dry needling, or injection-based approaches [35,36,37]. These findings suggest that rESWT represents an active therapeutic modality rather than a placebo-like intervention, although its superiority over other active treatments is not always demonstrated. Within this context, integrating shockwave therapy into multimodal rehabilitation strategies may represent a more effective approach than its isolated use.

The interpretation of these findings should also take into account the differences between focused and radial ESWT. Focused ESWT concentrates acoustic energy at a specific tissue depth, whereas radial ESWT generates a more superficial and dispersed pressure wave that distributes energy over a broader treatment area [20]. Focused shockwave therapy has produced the most consistent pooled evidence in meta-analyses, whereas radial rESWT remains less extensively studied. Jun et al. [37] report clearer superiority of focused devices in subgroup analyses. Nevertheless, the present findings indicate that radial shockwaves may still be clinically useful, particularly in superficial cervical myofascial structures where broader energy dispersion may be advantageous. Beneficial effects of rESWT have also been demonstrated in other musculoskeletal conditions. Lange et al. [38] observed significant improvements in pain and functional outcomes following rESWT in patients with acute low back pain, suggesting that the biological effects of radial shockwaves may extend across different musculoskeletal regions. In addition, previous studies have suggested that combining rESWT with other therapeutic approaches may enhance clinical outcomes. For example, Anwar et al. [30] reported greater improvements when rESWT was combined with ultrasound-guided trigger-point injection than when either intervention was applied alone.

Several biological mechanisms may contribute to the clinical effects of rESWT. Experimental and clinical evidence suggests that shockwave therapy can enhance local microcirculation, improve tissue oxygenation, and modulate nociceptive signaling within myofascial tissues [10,39]. Changes in neuromuscular activation patterns have also been reported after rESWT, including modifications in electromyographic activity that may reflect normalization of abnormal muscle recruitment associated with pain [40]. These biological responses may reduce peripheral sensitization and influence tissue mechanical properties, providing a plausible explanation for the concurrent improvements in muscle stiffness, pain intensity, and pressure pain threshold observed in the present study.

The follow-up findings should be interpreted cautiously. Although some outcomes remained favourable in the intervention group, between-group differences were generally smaller and less consistent at one month than those observed immediately after treatment. This pattern is consistent with previous literature, where short-term effects of rESWT are generally more pronounced than long-term ones [37]. Therefore, the present findings support the short-term effectiveness of adding rESWT to OMT, whereas conclusions regarding the persistence of treatment effects should be considered preliminary. Longer follow-up periods are needed to determine whether booster sessions or alternative treatment schedules are required to maintain clinical gains.

### 4.1. Study Limitations and Future Perspectives

Several limitations should be acknowledged. First, the sample size was relatively small considering the number of outcomes evaluated, and no formal a priori sample size calculation was performed. Although significant between-group differences were observed for several clinically relevant variables, the study was likely powered to detect only large effects in the primary outcome. Consequently, the possibility of both type I and type II errors cannot be completely excluded, particularly for secondary outcomes, and the findings should be interpreted as preliminary pending confirmation in larger randomized trials. Second, baseline differences were observed between groups, particularly in sex distribution and BMI. Although randomization was performed and baseline-adjusted analyses were applied, residual confounding cannot be completely excluded. Both sex and body composition may influence pain perception, pressure pain sensitivity, and muscle mechanical properties, and therefore may have partially contributed to the observed treatment effects. Larger randomized studies are needed to achieve greater baseline comparability and confirm the present findings. Third, SWE measurements were obtained from selected cervical regions and may not fully represent the global mechanical behavior of cervical musculature, and intra-rater reliability of SWE measurements was not formally evaluated. Fourth, the follow-up period was limited to one month, preventing conclusions regarding the long-term durability of treatment effects. Fifth, the lack of blinding of participants, therapists, and outcome assessors may have introduced performance and measurement bias. Sixth, participants were recruited using a consecutive sampling approach, which may increase the risk of selection bias. Seventh, the lack of control over pharmacological treatments represents a potential confounding factor. The use of analgesics, muscle relaxants, or other medications may have influenced pain-related outcomes and muscle mechanical properties, and therefore their contribution to the observed effects cannot be completely excluded. Finally, methodological heterogeneity remains a challenge in rESWT research, with substantial variability in device type, energy parameters, treatment frequency, and diagnostic criteria across studies.

Despite these limitations, the present findings suggest that combining rESWT with OMT may be associated with improvements in pain-related outcomes and cervical muscle mechanical properties. Future research should aim to clarify the role of elastography as a biomarker of treatment response in cervical myofascial pain. Larger randomized trials with longer follow-up periods are needed to confirm the durability of these effects, directly compare radial and focused shockwave devices, and explore whether baseline stiffness measured via elastography can help predict treatment response and guide personalized rehabilitation strategies.

### 4.2. Clinical Implications

The present findings have potential implications for clinical practice. The results suggest that rESWT could enhance the effects of OMT when both are integrated into a multimodal rehabilitation program for non-specific neck pain. In addition, the use of SWE may provide an objective method for assessing changes in muscle mechanical properties, complementing traditional clinical outcomes such as pain intensity or functional measures. Finally, rESWT represents a non-invasive intervention that may be incorporated into routine physiotherapy practice, particularly for superficial cervical myofascial structures.

## 5. Conclusions

The addition of rESWT to OMT was associated with greater improvements in cervical muscle stiffness, together with reductions in pain intensity and mechanical sensitivity in individuals with non-specific neck pain. These findings suggest that the use of rESWT may be a useful adjunct to manual therapy within multimodal rehabilitation programs. SWE may also provide a valuable objective tool for assessing treatment-related changes in cervical muscle mechanical properties. Further randomized trials with larger samples and longer follow-up are required to clarify the long-term clinical relevance of these findings and to optimize treatment protocols.

## Figures and Tables

**Table 1:** Immediate within-session changes in clinical, biomechanical, and elastography outcomes across the treatment sessions.

Variable	W1 CG	W1 IG	W2 CG	W2 IG	W3 CG	W3 IG	W4 CG	W4 IG
SWE (m/s)	−0.06 (0.35)	−1.55 (0.76) **	0.11 (0.40)	−1.18 (0.49) **	−0.01 (0.22)	−0.74 (0.39) **	−0.18 (0.39)	−0.59 (0.22) *
SWE (kPa)	−0.42 (8.11)	−35.70 (19.66) **	3.44 (10.46)	−25.18 (11.96) **	0.26 (5.84)	−13.88 (9.66) **	−4.11 (6.97)	−13.19 (7.42) *
PPT	1.34 (3.01)	8.68 (4.98) **	1.80 (3.02)	9.15 (5.97) *	2.48 (0.82)	7.07 (5.08) *	3.42 (2.51)	9.26 (5.74) *
VAS	−0.36 (1.49)	−2.71 (1.60) *	−0.57 (0.79)	−2.14 (1.57) *	−1.14 (0.69)	−1.79 (1.29)	−0.43 (0.79)	−1.43 (1.51)
ROM, flexion	−0.97 (6.12)	5.49 (8.08)	−1.81 (5.45)	3.54 (5.50)	1.44 (5.17)	5.92 (3.40)	2.75 (5.61)	3.90 (5.41)
ROM, extension	−1.02 (3.56)	2.95 (5.83)	3.38 (4.71)	3.04 (6.01)	0.57 (3.34)	6.93 (6.40)	0.10 (4.73)	2.02 (2.95)
ROM, right inclination	1.81 (1.43)	2.58 (2.50)	−0.28 (5.05)	2.38 (2.87)	−0.32 (1.44)	3.40 (6.47)	−0.23 (3.25)	4.64 (4.36) *
ROM, left inclination	0.23 (5.12)	3.77 (5.26)	1.39 (5.33)	4.98 (3.55)	1.88 (1.72)	4.08 (6.74)	1.43 (5.23)	2.04 (4.59)
ROM, right rotation	0.75 (2.31)	6.58 (4.72) *	1.04 (6.82)	2.72 (6.87)	1.43 (2.42)	4.73 (7.54)	1.88 (4.37)	3.92 (4.57)
ROM, left rotation	0.87 (4.12)	2.82 (4.92)	−0.39 (1.78)	0.94 (5.30)	1.09 (2.19)	5.50 (8.34)	−0.80 (4.27)	2.30 (3.37)
SES, right	−0.48 (1.81)	2.06 (1.93)	−0.66 (3.94)	−0.97 (2.02)	1.25 (2.32)	1.57 (1.44)	0.47 (1.62)	1.31 (2.69)
SES, left	0.46 (1.78)	0.65 (2.17)	−0.51 (1.63)	0.67 (2.20)	1.53 (1.77)	1.00 (2.29)	0.51 (2.51)	0.20 (3.06)

Values are expressed as mean (SD) of the change within each session (post-session minus pre-session values) during each treatment week. *p* values correspond to between-group comparisons of these within-session changes. CG: control group; IG: intervention group; SWE: shear wave elastography; PPT: pressure pain threshold; VAS: visual analogue scale; ROM: range of motion; SES: shoulder elevation strength. Statistical significance: * *p* < 0.05, ** *p* < 0.01.

**Table 2:** Changes from baseline to post-treatment by group and between-group comparison.

Variable	Δ CG	Δ % CG	Δ IG	Δ % IG	*p*
SWE (m/s)	−0.14 (0.56)	−1.7%	−1.70 (0.91)	−37.5%	0.005
SWE (kPa)	−2.10 (13.53)	2.4%	−38.74 (22.93)	−60.7%	0.007
PPT	5.29 (7.35)	13.3%	21.41 (14.50)	91.9%	0.064
VAS	−1.50 (1.38)	−35.0%	−3.64 (0.85)	−67.1%	0.009
ROM, flexion	1.99 (7.47)	3.0%	5.86 (7.52)	13.4%	0.306
ROM, extension	0.52 (6.86)	0.0%	10.89 (5.88)	21.4%	0.025
ROM, right inclination	4.90 (5.02)	23.8%	8.37 (6.22)	28.4%	0.160
ROM, left inclination	3.21 (6.02)	9.6%	7.92 (7.56)	21.9%	0.609
ROM, right rotation	2.99 (11.62)	5.2%	14.97 (9.76)	23.9%	0.055
ROM, left rotation	1.61 (4.82)	2.7%	7.27 (6.46)	10.6%	0.096
SES, right	2.17 (2.64)	9.6%	5.28 (3.34)	30.6%	0.097
SES, left	4.83 (5.32)	24.1%	3.30 (4.96)	19.7%	1.000

Values are expressed as mean (SD) change from baseline to post-treatment. Percentage change represents the relative change from baseline. *p* values correspond to between-group comparisons of the change scores.

**Table 3:** Persistence of treatment effects from post-treatment to 1-month follow-up.

Variable	Δ CG	Δ % CG	Δ IG	Δ % IG	*p*	Hedges g
SWE (m/s)	0.33 (0.30)	10.3%	−0.06 (0.51)	−1.8%	0.074	−0.869
SWE (kPa)	5.96 (9.91)	18.4%	0.29 (5.73)	3.7%	0.097	−0.655
PPT	−4.00 (4.82)	−11.0%	−0.25 (4.23)	1.6%	0.201	0.773
VAS	0.86 (0.90)	47.6%	0.07 (0.73)	26.8%	0.121	−0.897
ROM, flexion	−2.59 (6.18)	−4.0%	−1.50 (3.72)	−1.8%	1.000	0.200
ROM, extension	−4.33 (3.11)	−7.9%	0.70 (6.35)	1.9%	0.097	0.941
ROM, right inclination	1.09 (6.19)	6.1%	1.25 (4.16)	4.1%	0.371	0.028
ROM, left inclination	−2.86 (4.06)	−5.8%	−1.77 (3.75)	−3.0%	0.443	0.263
ROM, right rotation	−5.01 (7.04)	−6.3%	−1.34 (6.93)	−1.1%	0.608	0.493
ROM, left rotation	0.62 (3.86)	1.2%	−2.03 (5.16)	−2.7%	0.521	−0.545
SES, right	−0.69 (2.29)	−2.4%	−1.02 (2.81)	−2.5%	1.000	−0.121
SES, left	−0.93 (1.38)	−4.3%	0.85 (1.70)	5.2%	0.074	1.076

Values are expressed as mean (SD) change from post-treatment to 1-month follow-up. Percentage values represent relative changes. *p* values correspond to between-group comparisons of these changes. Effect sizes are presented as Hedges’ g.

**Table 4:** Adjusted treatment effects estimated via ANCOVA controlling for baseline values.

Variable	Beta	SE	95% CI		*t*	*p*	*p*FDR	R^2^ Adj
SWE (m/s)	−1.270	0.233	−1.783	−0.756	−5.444	<0.001	<0.001	0.706
SWE (kPa)	−27.976	5.566	−40.226	−15.725	−5.026	<0.001	<0.001	0.666
PPT	18.840	7.207	2.978	34.702	2.614	0.024	0.024	0.578
VAS	−2.127	0.713	−3.697	−0.557	−2.982	0.012	0.024	0.534
ROM, flexion	3.745	4.253	−5.615	13.105	0.881	0.397	0.397	0.557
ROM, extension	10.306	3.545	2.504	18.109	2.907	0.014	0.084	0.896
ROM, right inclination	3.814	3.241	−3.319	10.947	1.177	0.264	0.317	0.782
ROM, left inclination	4.699	3.894	−3.871	13.270	1.207	0.253	0.317	0.639
ROM, right rotation	12.097	5.807	−0.684	24.878	2.083	0.061	0.183	0.304
ROM, left rotation	5.646	3.334	−1.692	12.984	1.694	0.118	0.236	0.818
SES, right	3.857	1.698	0.119	7.594	2.271	0.044	0.088	0.830
SES, left	−1.508	2.818	−7.711	4.694	−0.535	0.603	0.603	0.337

SWE: shear wave elastography; PPT: pressure pain threshold; VAS: visual analogue scale; ROM: range of motion; SES: shoulder elevation strength.; *p*FDR: Benjamini–Hochberg false discovery rate adjusted *p*-values calculated within outcome domains.

## Data Availability

The datasets used and/or analysed during the current study are available from the corresponding author upon reasonable request.

## Abbreviations

rESWT	Radial extracorporeal shockwave therapy
OMT	Orthopedic manual therapy
SWE	Shear wave elastography
VAS	Visual analogue scale
PPT	Pressure pain threshold
ROM	Range of motion
SES	Shoulder elevation strength
CG	Control group
IG	Intervention group
RAPA	Rapid Assessment of Physical Activity
